# Microbial Fermentation as a Tool to Improve the Antioxidant and Functional Value of Milk Products

**DOI:** 10.3390/foods15061024

**Published:** 2026-03-15

**Authors:** Sion Seol, JuDong Yeo

**Affiliations:** Department of Food Science and Biotechnology of Animal Resources, Konkuk University, Seoul 05029, Republic of Korea; seolsion@konkuk.ac.kr

**Keywords:** fermentation, dairy food, metabolite, antioxidant, bioactives

## Abstract

Microbial fermentation is attracting attention as a key process in reconstructing the profile of functional components in foods. This review summarizes the main mechanisms by which microbial fermentation generates antioxidants and functional compounds in fermented dairy products. In particular, we focus on (i) the production of bioactive peptides driven by fermentation-induced proteolysis, (ii) modulation of reactive oxygen species (ROS) homeostasis associated with shifts in metabolite composition, and (iii) the remodeling of organic acids, fatty acids, and other low-molecular-weight metabolites. We also discussed an analytical framework for evaluating antioxidant function in various analytical methods such as the 2,2′-azino-bis(3-ethylbenzothiazoline-6-sulfonic acid) (ABTS) assay, 2,2-diphenyl-1-picrylhydrazyl (DPPH) assay, and ferric reducing antioxidant power (FRAP) assay, as well as cell-based measurements of reactive oxygen species (ROS)/nitric oxide (NO) and determining oxidative damage in animal models. Overall, the antioxidant functionality of fermented dairy products should be understood not merely as an increase in radical-scavenging capacity but as the outcome of fermentation-driven molecular remodeling and physiological regulatory effects. This review defines fermented dairy products as functional foods, highlighting the need for an omics-based approach in future fermented food studies.

## 1. Introduction

Oxidative stress is a major contributing factor to the development of chronic diseases such as diabetes, cancer, and cardiovascular disease, primarily due to the disruption of the antioxidant defense system of the body by reactive oxygen species (ROS) [1]. Along with the growing awareness of this concept, the importance of antioxidant foods as a dietary strategy to regulate excessive oxidative stress and maintain redox homeostasis in the body continues to be increasingly recognized.

Microbial fermentation is understood as a process that reshapes the chemical structures of antioxidants and functional constituents within the food matrix through microorganism-associated enzymatic systems, particularly extracellular enzymes, thereby altering the overall antioxidant profile and potentially improving bioavailability [2]. These fermentation-driven changes can be broadly explained by two major routes: (i) the generation of bioactive peptides via proteolysis and (ii) the microbial biotransformation of polyphenols into lower–molecular-weight derivatives with enhanced antioxidant potential. First, fermentation-derived peptides may directly scavenge ROS through the reductive capacity (electron-donating properties) of specific amino-acid residues and, in parallel, activate the Nrf2 signaling pathway, leading to increased expression of antioxidant enzymes such as SOD and GPx [3]. Second, polyphenols can undergo deglycosylation and depolymerization during fermentation, yielding smaller molecules that may exhibit stronger radical-scavenging activity [4]. Moreover, phenolic metabolites (e.g., ferulic acid and caffeic acid) have been proposed to further stimulate the Nrf2 pathway, thereby promoting antioxidant enzyme induction [5]. The observation that fermented tea, cocoa, and fruit wines often exhibit stronger antioxidant effects than their unfermented counterparts is consistent with this mechanism. Collectively, these findings suggest that fermentation does not merely increase the abundance of bioactive compounds; rather, it structurally optimizes peptides and polyphenol-derived metabolites to converge on shared antioxidant pathways, thereby amplifying protection against oxidative stress. However, improvements in in vitro antioxidant assays do not necessarily translate into clinical efficacy in humans, and the level of supporting evidence varies substantially across fermentation systems and study designs.

Most previous reviews have primarily focused on functional outcomes or individual mechanisms, while the emerging role of advanced analytical technologies and omics-based approaches in elucidating fermentation-derived bioactive compounds has received comparatively less integrated discussion. Advanced analytical approaches for fermented foods increasingly rely on omics technologies. Metabolomics (NMR- and MS-based) is the most widely used, enabling broad qualitative and quantitative profiling of metabolites and pathway-level interpretation in complex fermented food matrices. NMR-based metabolomics allows single-experiment, high-throughput screening and strain comparison, especially when combined with multivariate statistics to distinguish metabolic phenotypes and taxa [6]. MS-based metabolomics, particularly when coupled with GC–MS, LC–MS, or CE–MS and high-resolution analyzers, provides sensitive, untargeted coverage for tracking fermentation dynamics, discovering bioactives, and assessing authenticity [7]. Metagenomics characterizes the taxonomic composition and functional potential of microbial communities in fermented foods based on community DNA [8]. Metatranscriptomics complements this by profiling community mRNA, revealing which genes are actively expressed during fermentation and thus indicating functional activity [9]. Proteomics further captures dynamic changes in protein abundance and modifications, providing insight into functional protein regulation over time. Multi-omics approaches integrate these layers to jointly link microbial composition, gene expression, protein function, and metabolite production [8]. This integration is shifting fermented food research from empirical observation toward data-driven precision fermentation and process optimization. MS-based analytical platforms were used in the majority of the studies included in this review, highlighting their central role in the characterization of fermentation-derived bioactive compounds and compositional changes in fermented dairy products.

**Figure 1:**
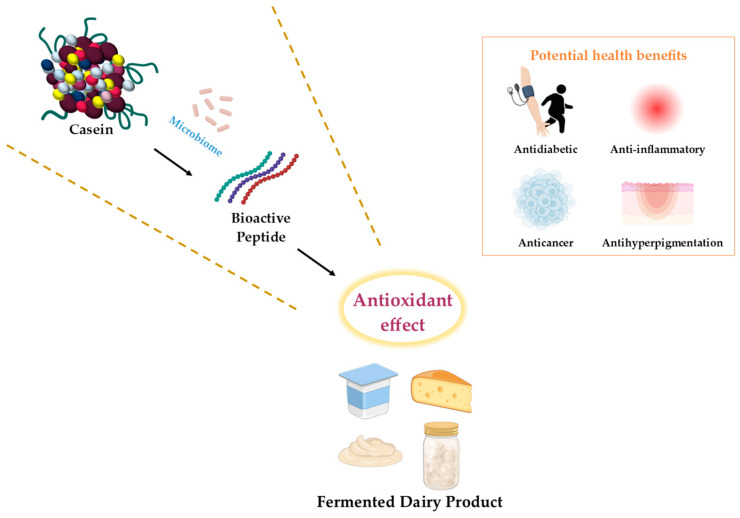
Schematic overview of bioactive peptide generation during dairy fermentation and its associated antioxidant effects. Schematic illustration of bioactive peptide generation from casein through microbial activity and its association with antioxidant effect in fermented dairy products. Arrows indicate the proposed flow from casein and the microbiome to bioactive peptide formation and antioxidant effect. Dotted lines indicate conceptual boundaries, and the framed panel summarizes the potential health benefits, including antidiabetic, anti-inflammatory, anticancer, and antihyperpigmentation activities.

The functional properties of fermented milk can vary substantially depending not only on the microbial species used but also on characteristics at the strain level. Even microorganisms belonging to the same species may differ in proteolytic capacity, exopolysaccharide (EPS) production, fatty acid metabolism, and the generation of bioactive peptides, and these differences may lead to distinct functional properties and health-related effects in fermented milk products. However, because such strain-specific effects and related mechanisms are often reported across individual studies, it is not easy to compare representative cases at a glance. Accordingly, in this review, representative strain examples from studies discussed in later sections were selectively summarized in Table 1 according to their strain-specific functional effects and proposed mechanisms. The conceptual relationships between the major bioactive components formed during fermentation, their antioxidant effects, and their potential health benefits are illustrated in Figure 1.

## 2. Methods

This manuscript was conducted as a narrative review employing a structured literature search to minimize selection bias. Literature searches were performed in PubMed, Scopus, and Google Scholar covering the period from January 2016 to 13 February 2025. Studies were included if they (i) investigated fermented dairy products, (ii) evaluated antioxidant-related outcomes using chemical assays and/or cellular, animal, or human models, and (iii) clearly reported fermentation conditions and/or microbial strains. Conference abstracts, non-English publications, and studies that did not report antioxidant-related outcomes were excluded. Two authors independently screened titles and abstracts, followed by full-text assessment for eligibility. Disagreements were resolved through discussion. Study quality was qualitatively assessed based on the clarity of the experimental design, the use of appropriate controls, the adequacy of statistical reporting, and the transparency of reported fermentation variables. Risk-of-bias considerations (e.g., controls, potential confounding, and selective reporting) were assessed qualitatively, and evidence was interpreted accordingly.

## 3. Microbial Fermentation in Dairy Foods

The composition of microorganisms involved in dairy fermentation is a key factor that determines the chemical composition, physicochemical properties, and functional characteristics of fermentation products. Fermented dairy products are shaped not merely by the metabolic activity of a single microorganism but instead exhibit complex fermentation mechanisms driven by microbial communities that shift across fermentation stages and by the metabolic interactions among these microorganisms [18]. Such temporal shifts in microbial composition and functional differentiation induce stepwise transitions in protein, lipid, and carbohydrate metabolism, rather than merely acid production, thereby shaping the distinctive structural and functional characteristics of fermented dairy products.

### 3.1. Yogurt Fermentation

Yogurt fermentation is a representative fermentation system described as a symbiosis between *Streptococcus thermophilus* and *Lactobacillus delbrueckii* subsp. *bulgaricus* [19]. The two strains perform distinct metabolic roles throughout fermentation, and their functional contributions vary depending on the fermentation stage. In the early phase of fermentation, *S. thermophilus* utilizes lactose to produce lactic acid, thereby lowering the pH of the fermentation environment and establishing acidic conditions suitable for yogurt fermentation [20]. This process provides a favorable environment for the growth and metabolism of *L. delbrueckii* subsp. *bulgaricus*. Therefore, as fermentation proceeds, the proteolytic activity of *L. delbrueckii* subsp. *bulgaricus* increases, leading to the release of amino acids and peptides that serve as nitrogen sources. These compounds support the growth and metabolic activity of fermenting microorganisms, including *Streptococcus thermophilus*, thereby strengthening interdependent metabolic relationships among them. This functional differentiation and stepwise interaction indicate that yogurt fermentation is a biotransformation process that goes beyond lactic acid production and is accompanied by the activation of amino acid metabolic pathways [21]. As a result, the formation of the characteristic yogurt gel structure and viscosity is induced, and the metabolite composition of the fermented milk also changes over time [22].

### 3.2. Cheese Fermentation

Cheese fermentation is a process that shows clear changes in microorganisms from the initial fermentation stage to the aging stage. Starter lactic acid bacteria lower the pH by converting lactose to lactic acid in the early fermentation stage, helping to solidify and form a cheese matrix. It stabilizes the structure of cheese and supports the settlement of microorganisms. As aging continues, the number of starter strains relatively sensitive to acidic and high salt conditions gradually decreases, but non-starter lactic acid bacteria such as *Lacticaseibacillus casei* group become dominant. This shift in microbial composition indicates that the cheese fermentation mechanism transitions from being centered on lactose metabolism to being centered on protein and lipid degradation [23]. During ripening, microorganisms decompose casein and milk fat to produce low-molecular metabolites such as peptides, free amino acids, and fatty acids, contributing to the taste and texture of cheese. Therefore, cheese fermentation constitutes a fermentation system in which the temporal succession of microbial communities is organically coupled with the consequent shifts in metabolic pathways [24].

### 3.3. Sour Cream Fermentation

For sour cream fermentation, *Streptococcus thermophilus* and *Lactobacillus delbrueckii* are used in the fermentation process. As sour cream ferments, the relative proportion of *L. delbrueckii*, which is highly resistant to acidic conditions, increases. This plays an important role in the sour cream fermentation mechanism. An acidic environment is created by lactic acid production, and the lipid enzymes in the cream are hydrolyzed to form free fatty acids. In addition, fermentation proceeds, and these free fatty acids undergo oxidation and conversion reactions to produce volatile compounds such as aldehyde, ketone, alcohol, and ester, contributing to the sour and creamy flavor unique to sour cream. Thus, sour cream fermentation can be described as a stepwise process in which lactic acid production and microbial compositional shifts sequentially drive lipid breakdown and flavor formation [25].

### 3.4. Kefir Fermentation

**Figure 2:**
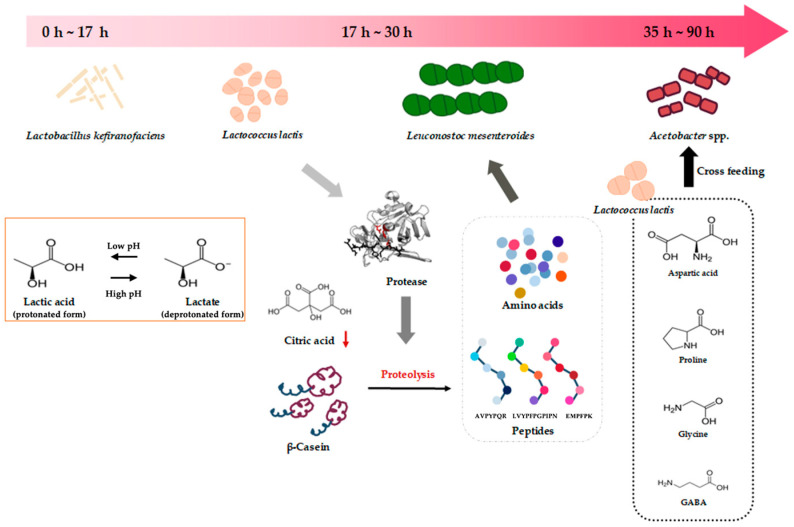
Shifts in microbial niches and metabolic changes during kefir fermentation. The top horizontal arrow indicates the fermentation timeline from the early to late phase. Different colored microbial symbols represent distinct microbial taxa, and the arrows indicate the proposed succession of microbial activity and metabolic flow. The framed panel illustrates the lactic acid/lactate equilibrium associated with pH change during early fermentation. Proteolysis of β-casein by microbial proteases contributes to the release of free amino acids and peptides, shown in the central dotted box. In the later phase, metabolites produced by *Leuconostoc mesenteroides*, including aspartic acid, proline, glycine, and γ-aminobutyric acid (GABA), may be utilized in cross-feeding interactions that support the growth of *Acetobacter* spp., as indicated in the right dotted box.

Figure 2 shows alterations in microbial composition and the changes in metabolites during kefir fermentation. Kefir fermentation constitutes a complex microbial ecosystem where lactic acid bacteria and yeast coexist. This leads to greater metabolic diversity than fermentation by a single strain of lactic acid bacteria. In the early phase of fermentation, a lactic acid bacterial consortium including *Lactococcus lactis* utilizes lactose as a primary carbon source to produce lactic acid, resulting in a decrease in pH and establishment of the fermentative environment [26]. Concurrently, proteolysis is initiated by protease activities within the early microbial community, and the resulting pool of free amino acids and peptides is subsequently utilized to support the growth and metabolism of other community members, including *Leuconostoc mesenteroides* [27]. As fermentation progresses, citrate is gradually depleted, while proteolysis continues, leading to an increase in the level of free amino acid and peptide pools. During this process, β-casein–derived bioactive peptides such as AVPYPQR, LVYPFPGPIPN, and EMPFPK have been detected in fermented kefir products and have been reported to be associated with physiological functions, including angiotensin-converting enzyme (ACE) inhibition [28]. In the late phase of fermentation, *Acetobacter* spp. have been suggested to preferentially grow by utilizing aspartic acid, proline, glycine, γ-aminobutyric acid (GABA), and other low-molecular-weight metabolites produced by lactic acid bacteria and yeasts [29]. Notably, the stable microbial community formed within kefir grains through interspecies interactions maintains enzymatic activity throughout fermentation, thereby contributing to the chemical diversity of the fermentation products and enhancing their functional properties [30].

## 4. Changes in Antioxidant and Functional Properties of Dairy Foods During Fermentation by Focusing on Peptides

Changes in antioxidant capacity and functional properties of fermented dairy products, as summarized in Table 2, vary widely depending on the type of dairy matrix, the microorganisms involved, and the analytical methods used.

### 4.1. Yogurt Fermentation and Antioxidant-Related Effects

Yogurt fermentation has been associated with a concerted attenuation of systemic oxidative stress across diverse animal models. In a high-fat diet (HFD)–induced model, yogurt supplementation (5% *w*/*w*, 8 weeks) significantly reduced plasma and hepatic malondialdehyde (MDA), NO, and apoptosis (APOP) levels [31]. Similarly, in an STZ-induced diabetes model, MDA increased by approximately 104.6% relative to controls (42.1 ± 2.1 vs. 29.31 ± 1.9 nmol/g tissue), whereas GSH decreased by approximately 35.4% (9.3 ± 0.5 vs. 14.4 ± 1.0 mg/g tissue). Administration of yogurt fermented with *L. rhamnosus* KU985439 partially but significantly mitigated these alterations, reducing MDA by 71.82% and restoring GSH by 40.28% relative to the diabetic control group, thereby suggesting a partial reinforcement of the endogenous redox-buffering system [10]. Notably, the observation that comparable antioxidative effects were evident under distinct pathological contexts, HFD-induced metabolic stress and STZ-induced diabetes, suggests that the benefits of fermented yogurt may not be restricted to a single model but rather reflect broader redox-regulatory mechanisms. Overall, the available evidence supports that yogurt fermentation contributes to systemic redox homeostasis through a dual mode of action, encompassing both a reduction in oxidative damage markers and an enhancement of endogenous antioxidant capacity.

### 4.2. Metabolic Alterations in Mixed-Culture Fermented Milk

Milk fermentation by complex microbial consortia, as exemplified by kefir and koumiss, drives a coherent and coordinated remodeling at both the peptide and metabolite levels. One characteristic feature reported in kefir fermentation is the generation of β-casein–derived phosphorylated peptides through microbial proteolysis. Across studies, these phosphopeptides are enriched in the canonical calcium-binding motif (SerP–SerP–SerP–Glu–Glu). Notably, among 73 phosphopeptides identified by LC–ESI–QTOF–MS/MS, 71 contained this motif and 79% were derived from β-casein, potentially suggesting functional links to mineral chelation and improved bioavailability. Importantly, this structural motif may confer dual physiological potential, encompassing modulation of mineral metabolism and contribution to antioxidant activity via peptide-mediated redox interactions [13]. Beyond biochemical characterization, peptide-enriched fractions of kefir have demonstrated functional activity across multiple biological systems. In vitro, fractions with higher peptide content exhibited greater ferric-reducing antioxidant power, and the <10 kDa peptide fraction showed significantly higher FRAP activity and acetylcholinesterase inhibitory capacity than the other fractions (*p* < 0.05). In vivo, in a Drosophila melanogaster Alzheimer-like model, treatment with kefir fractions at 0.25 mg/mL improved climbing ability and, after 10 days, reduced β-amyloid content and neurodegeneration indices compared with untreated flies, while only the <10 kDa fraction at 0.25 mg/mL significantly decreased acetylcholinesterase activity. Collectively, these findings support a peptide-mediated neuroprotective effect in an AD-like context, potentially involving antioxidant and cholinergic pathways rather than a single redox-dependent mechanism [33]. Taken together, kefir fermentation can be interpreted as a process that promotes selective peptide release accompanied by functionally relevant antioxidant effects. In parallel with peptide remodeling, mixed-culture fermentation induces systematic metabolic reprogramming, as demonstrated in koumiss. Untargeted metabolomics revealed clear separation between unfermented milk and koumiss, supported by OPLS-DA model parameters (R^2^ = 0.862, Q^2^ = 0.848), indicating broad and consistent shifts in metabolic architecture [34]. Among 166 differential metabolites (61 upregulated, 105 downregulated), fermentation was associated with increased levels of pyruvate, γ-aminobutyric acid (GABA), and acetoacetate, alongside decreases in γ-linolenic acid and bile-related metabolites (all *p* < 0.05, VIP > 1). Pathway enrichment highlighted enhanced amino acid metabolism and restructuring of redox-related metabolic pathways, with 7 core pathways significantly upregulated among 149 mapped pathways [34]. Certain lactic acid bacteria can convert linoleic acid to conjugated linoleic acid (CLA), which may be interpreted as part of the lipid remodeling that occurs during fermentation. Owing to its two conjugated double bonds, CLA exhibits distinctive chemical behavior in oxidative reactions, and multiple studies have reported radical-scavenging activity and antioxidant capacity for CLA. Notably, CLA has been described as one of the major antioxidant-active constituents within milk fat, and potential interactions with lipophilic antioxidants such as α-tocopherol and β-carotene have also been proposed [38].

Overall, evidence from kefir and koumiss supports the interpretation that mixed-culture fermentation drives a dual remodeling process: (i) selective release of structurally functional peptides via targeted proteolysis, and (ii) reprogramming of low-molecular-weight metabolite networks linked to redox homeostasis. These coordinated molecular changes may represent not merely compositional shifts, but a mechanistic basis for the systemic antioxidant and physiological effects attributed to fermented dairy products.

### 4.3. Metabolic Alterations in Probiotic Fermented Milk Products Using Specific Strain

Studies on probiotic fermented milk products manufactured using selected strains have reported functionality-associated metabolic shifts based on untargeted UPLC–MS/MS metabolomics. Milk fermented with *Lactiplantibacillus plantarum* T1 or CSK exhibited increased scavenging activities against ABTS, DPPH, and hydroxyl radicals. Specifically, ABTS scavenging rates reached 77.49% (T1) and 71.52% (CSK), DPPH scavenging rates were 42.11% (T1) and 38.12% (CSK), and hydroxyl radical scavenging rates were 49.44% (T1) and 41.46% (CSK). Untargeted metabolomics identified 434 (T1) and 443 (CSK) metabolites across positive and negative ion modes, predominantly comprising lipids and lipid-like molecules (27.27%), organic acids and derivatives (20.25%), and organic heterocyclic compounds (16.53%) [36]. The authors suggested that phenotypic differences between strains were reflected in distinct metabolite profiles; notably, *L. plantarum* CSK produced substantially higher levels of fumaric acid, a metabolite with reported antioxidant properties. In addition, both strains significantly increased organic acids (upregulated species: 62 for T1 and 41 for CSK) and amino acids (36 and 35 differential features, respectively) relative to unfermented milk [36]. Among the diverse metabolic products generated by lactic acid bacteria (LAB) during fermentation, exopolysaccharides (EPS) represent a prominent class of postbiotic metabolites secreted into the fermentation matrix [39]. The biosynthesis of EPS is directly coupled to carbohydrate utilization and sugar metabolic flux: dietary sugars are first transported into the cell and phosphorylated, after which intracellular sugar nucleotides, including UDP-glucose, UDP-galactose, and dTDP-rhamnose, are formed. These activated precursors are subsequently assembled into repeating unit structures, anchored to membrane-associated undecaprenol diphosphate carriers, and translocated to the extracellular space via Wzx flippase. Accordingly, the metabolic fate of carbohydrates present as fermentation substrates is not limited to energy generation but extends to the synthesis and secretion of high-molecular-weight polysaccharide polymers. This implies that fermentation-driven redistribution of carbon flux toward EPS biosynthesis may represent a mechanistic basis for the observed increases in antioxidant capacity in fermented dairy products, as the accumulation of EPS in the fermentation matrix has been associated with free radical-scavenging and antioxidant activities. It should be noted, however, that certain studies have raised concerns regarding the potential cytotoxic and carcinogenic properties of EPS, warranting cautious interpretation when attributing functional benefits to this class of metabolites [40]. On this basis, they concluded that *L. plantarum* fermentation can enhance antioxidant activity and improve the nutritional quality of fermented milk. Similarly, UPLC–Q Exactive–MS (UPLC–QE–MS)–based metabolomics was used to track metabolite dynamics during milk fermentation by *Lacticaseibacillus paracasei* PC-01 and *Bifidobacterium adolescentis* B8589 [35]. Metabolite composition changed most prominently during the early fermentation phase (0–36 h), during which 304 differential metabolites (151 upregulated, 153 downregulated) were identified, whereas only 110 and 36 differential metabolites were detected in the 36–60 h and 60–72 h intervals, respectively, indicating that metabolic remodeling was most active at the onset of fermentation. GABA increased continuously over time and reached its highest level at the end of fermentation (72 h), exhibiting a correlation with the progressive acidification of fermented milk. Based on these findings, the authors proposed that fermentation-driven metabolic changes may be potentially linked to improved antioxidant capacity in vivo. In addition, succinic acid, a signaling metabolite implicated in oxidative stress and inflammatory regulation, was significantly upregulated at 36 h (fold change > 2, *p* < 0.05), whereas fumaric acid, an oxidative downstream product of succinate, decreased throughout fermentation, suggesting preferential consumption of fumaric acid by probiotic strains. These metabolite shifts were associated with pathway-level remodeling involving the TCA cycle, glutamate/GABA-related metabolism, and fatty acid metabolism. Nine stage-specific differential biomarkers (pyruvic acid, succinic acid, fumaric acid, L-glutamic acid, GABA, capric acid, oleic acid, palmitic acid, and stearic acid) were identified as major contributors to significantly enriched pathways [35]. The authors discussed that such pathway-level reprogramming may contribute to the putative beneficial properties of probiotic fermented milk products.

### 4.4. Antioxidant and Bioactivity of Peptides from Fermented Dairy Products

Across a broad range of fermented dairy matrices, beyond conventional milk-based products, peptides generated via microbial proteolysis during fermentation have been consistently reported and are frequently linked to antioxidant and anti-inflammatory functions. A recurring pattern across studies is the selective accumulation of low-molecular-weight peptides derived from β-casein. In a proteomics/peptidomics analysis of goat milk kefir using EASY-nLC–Orbitrap MS/MS, a total of 97 peptides (68 unique sequences) were identified from three kefir samples, with β-casein confirmed as the most extensively hydrolyzed protein. Amino-acid sequence coverage was 31.08% (GKCN), 49.55% (GKEU), and 22.97% (GKUSA). Among the major products were β-casein–derived peptides including EMPFPK (residues 108–113), reported to exhibit ACE-inhibitory activity, and VLPVPQK (residues 170–176), reported to possess antioxidant activity. Notably, β-casein coverage remained below 50% in all samples, suggesting that the proteolytic capacity of kefir-grain consortia is insufficient for complete protein digestion. Accordingly, the observed peptide profile is more appropriately interpreted as a set of “selectively accumulated” peptides rather than terminal products of exhaustive proteolysis [32].

This trend was similarly observed in other fermented dairy products. In Binglangjiang buffalo fermented milk (BBFM), peptidomics/proteomics analysis using LC–MS/MS identified 769 peptides from an ultrafiltration fraction (<3000 Da), of which 84.01% fell within the 1000–2000 Da range. Functional screening in an LPS-stimulated RAW264.7 macrophage model led to the identification of a novel peptide, GG13 (GPGAPADPGRPTG; molecular weight = 1149.56 Da). Basal NO levels in unstimulated RAW264.7 cells remained low (0.22 μmol/L) but increased significantly to 2.03 μmol/L following LPS stimulation (*p* < 0.001). GG13 exerted concentration-dependent effects and, at 200 μg/mL, inhibited NO secretion by 68.81% and TNF-α secretion by 40.25% (*p* < 0.001), while maintaining hemolytic activity below 2% across all tested concentrations, supporting its safety profile Because inflammatory signaling is frequently coupled with ROS activation, subsequent proteomics analysis further showed that GG13 significantly downregulated 27 inflammation-related proteins, including STAT1, NOS2, COX2, and CD40 (fold change < 0.67, *p* < 0.05), and was implicated in 115 significantly enriched KEGG pathways [37]. Consistently, LC–MS/MS analyses of cheese fractions repeatedly detected β-casein–derived antioxidant peptides such as AVPYPQR and VLPVPQK; EMPFPK was reported to display bradykinin-like activity, whereas AMKPWIQPK showed ACE-inhibitory activity [11]. Notably, these peptides commonly contain amino-acid residues associated with radical-scavenging potential (e.g., methionine, histidine, and proline), providing sequence-level plausibility for antioxidant mechanisms.

Taken together, the selective accumulation of low-molecular-weight β-casein–derived peptides across kefir, buffalo fermented milk, and cheese supports the view that fermentation establishes a proteolysis-driven functional axis centered on specific bioactive sequences. The observation that β-casein hydrolysis coverage in kefir remains below 50% and that 84.01% of BBFM peptides cluster within the 1000–2000 Da range further indicates that fermentation-derived peptide profiles are structurally biased toward smaller, functionally potent products. These peptides may contribute to the mitigation of oxidative stress and related physiological benefits not only through direct radical-scavenging activity but also via ACE inhibition and quantitative suppression of inflammatory mediators such as NO and TNF-α.

### 4.5. Comparative Patterns Across Fermentation Systems and Evidence Strength

The reviewed studies are fundamentally difficult to compare quantitatively because they differ in substrates, starter compositions, and fermentation conditions (temperature/time). Still, limited comparisons are possible within the same assay: A LAB–yeast consortium (*L. fermentum* KGL4/S. *cerevisiae* WBS2A; 30 °C, 48 h) increased ABTS activity from 1.70% to 40.08% (≈23.6-fold), ~5–6% higher than a single-LAB fermentation (*L. plantarum* KGL3A; 34.5%), whereas a Brazilian kefir–sheep milk system matured at 4 °C for 28 days reached 41.49%, implying that fermentation strategy may affect antioxidant gains as strongly as starter composition [14,33]. Heterogeneity is greater in in vivo studies (models, durations, and reporting), limiting effect-size comparisons. Moreover, assay choice constrains interpretation because FRAP, DPPH/ABTS, and ORAC capture different mechanisms; several studies used proteomics/metabolomics or cell-based ROS endpoints without parallel measurement of standard radical-scavenging assays. Therefore, despite indications of strain-dependent synergy, standardized head-to-head studies controlling substrate and fermentation parameters and applying DPPH, ABTS, FRAP, and ORAC in parallel are needed. Moreover, potential discrepancies between such laboratory-scale fermentations and industrial manufacturing—including larger fermentation volumes, different mixing and oxygen transfer, diverse cooling profiles, and extended storage and distribution chains—are rarely addressed explicitly in the current literature.

## 5. Antioxidant Capacity of Fermented Dairy Products

### 5.1. Chemical Antioxidant Capacity

Antioxidant capacity in fermented dairy products can be evaluated through in vitro chemical assays and in vivo systems. Among these, in vitro assessments commonly employ radical-scavenging assays such as ABTS, DPPH, and hydroxyl radical scavenging activity, as well as reducing power-based methods such as FRAP. Antioxidant capacity measurements (e.g., DPPH, ABTS, FRAP) represent chemical reactivity under controlled laboratory conditions and should be interpreted separately from biological effects demonstrated in cellular, animal, or human studies. ABTS assay measures antioxidant activity by monitoring the decrease in absorbance at 734 nm of the ABTS radical cation, which is generated via oxidation by potassium persulfate, thereby reflecting radical scavenging driven by electron and/or hydrogen donation [41]. However, the ABTS assay is very sensitive to conditions like pH and how long the reaction runs. Because of this, the measured antioxidant activity might not only reflect the original compound’s effect, but also the effects of new by-products that are formed during the test. Accordingly, particularly in interpreting the antioxidant activity of peptides, it is necessary to distinguish whether the observed effects arise from the peptide itself or from oxidation-derived products generated during the reaction [42]. Similarly, 1,1-diphenyl-2-picrylhydrazyl (DPPH) assay is based on the neutralization of a stable purple radical through acceptance of an electron or hydrogen atom from antioxidant compounds, accompanied by a color change toward yellow; the resulting decrease in absorbance is used to estimate radical-scavenging capacity [43,44]. In contrast, the FRAP assay quantifies reducing capacity based on the chemical reaction from Fe^3+^ to Fe^2+^ mediated by antioxidants, and thus predominantly reflects a single-electron transfer mechanism, with antioxidant activity evaluated via the absorbance of the generated Fe^2+^ species. Nevertheless, since FRAP does not capture hydrogen atom transfer (HAT)–based reactions, it has inherent limitations in comprehensively assessing the full spectrum of antioxidant mechanisms [45]. Taken together, ABTS and DPPH should be interpreted as indices that assess antioxidant activity primarily through scavenging of stable free radicals, whereas FRAP should be understood as an index reflecting electron-donating capacity via metal-ion reduction. Because these assays capture distinct mechanistic dimensions of antioxidant action, the antioxidant properties of fermented dairy products are more appropriately interpreted by integrating multiple complementary indices rather than relying on a single in vitro endpoint. In vitro antioxidant assays differ substantially from in vivo conditions in terms of reaction environments and radical targets (e.g., ABTS and DPPH employ relatively stable artificial radicals), and the applied concentrations and reaction times (minutes to hours) deviate markedly from the physiological timescales of short-lived radicals in in vivo (e.g., ${HO}^{•}$), thereby limiting their biological relevance [46]. Accordingly, no single in vitro assay can fully recapitulate natural oxidative processes occurring in vivo, as each method measures only a limited aspect of antioxidant capacity. In particular, because DPPH and ABTS rely on physiologically irrelevant artificial radicals, these assays are inherently restricted to evaluating direct antioxidant–radical interactions [47].

### 5.2. Cellular Antioxidant Effects

Accordingly, recent studies have sought to address the limitations of in vitro assays by indirectly validating the cellular antioxidant potential of fermented milk–derived constituents using LPS-stimulated RAW264.7 macrophages, where reductions in ROS or NO production are employed as readouts. Such cell-based approaches suggest that bioactives in fermented milk may modulate oxidative stress under inflammatory stimulation beyond simple radical scavenging. Microbial fermentation selectively releases peptides encrypted within milk proteins via microorganism-specific proteolytic systems, and the resulting peptides often exhibit broader functional potential in terms of sequence diversity and bioactivity than products generated by simple enzymatic hydrolysis [48]. The peptides formed during fermentation are therefore not merely random degradation products of milk proteins; rather, they can be explained by quantitative process-associated changes, including reductions in apparent protein molecular weight and the selective accumulation of low-molecular-weight peptides. In this regard, High-molecular-weight proteins decreased during fermentation and storage using SDS-PAGE and size-exclusion chromatography, concomitant with the accumulation of low-molecular-weight peptides, particularly within the sub-3 kDa range [49]. Collectively, these observations suggest that the peptide diversity observed in fermented dairy products reflects fermentation-induced alterations in protein structure and selective peptide persistence, rather than simply the extent or intensity of hydrolysis. The alteration of pH modulates protein structural state (e.g., oligomerization), charge distribution, and hydrogen-bonding capacity, thereby regulating binding affinity and stability toward small molecules. β-lactoglobulin (β-LG)–acrylamide (ACR) interactions showed a higher binding constant (K_a_) at pH 7.0 than at pH 3.0, indicating that pH directly governs the strength of protein–ligand interactions [50]. They further reported a more negative ΔG at pH 7.0, consistent with increased binding stability, and showed that β-LG exists predominantly as a monomer at pH 3.0 but as a dimer at pH 7.0. Meanwhile, organic acids are not necessarily potent radical scavengers; however, under specific conditions they may function as modulators that enhance antioxidant effects through synergistic interactions with non-phenolic antioxidant compounds [51]. Low-molecular-weight organic acids, including citric acid, were reported to alleviate oxidative stress under heavy metal stress conditions by increasing the activities of antioxidant enzymes (SOD, CAT, and POD) and reducing the levels of lipid peroxidation markers [52]. This is included in the mechanisms for indirectly relieving oxidative stress through endogenous antioxidant defense.

### 5.3. In Vivo Biological Relevance

Furthermore, oxidative damage in animal models has been evaluated by measuring TBARS, a widely used indicator of lipid peroxidation [53]. In parallel, changes in endogenous antioxidant defense systems, such as superoxide dismutase (SOD), catalase (CAT), glutathione peroxidase (GSH-Px), and glutathione (GSH), are commonly assessed as biomarkers of antioxidant status [54]. Mechanistically, SOD acts as an antioxidant enzyme by catalyzing the conversion of superoxide anion (O_2_^−^) into hydrogen peroxide (H_2_O_2_), with the release of molecular oxygen [55]. In turn, CAT decomposes hydrogen peroxide into water and oxygen, thereby maintaining cellular redox balance [56]. GSH is widely recognized as a central endogenous antioxidant that protects cells from oxidative stress by maintaining intracellular redox homeostasis and directly scavenging reactive oxygen species [57]. Modulation of these endogenous antioxidant defenses (e.g., SOD, CAT, GSH-Px, and GSH) provides a rationale for interpreting the antioxidant effects of fermented milk beyond in vitro radical-scavenging capacity, extending the interpretation toward regulation of organism-level defense capacity. Notably, when indices of oxidative damage such as TBARS or MDA are presented alongside biomarkers of endogenous antioxidant defenses, the linkage between reduced oxidative damage and restored defense systems becomes more explicit, thereby strengthening the biological plausibility of functional claims.

However, the antioxidant functionality of fermented dairy products cannot be adequately interpreted solely on the basis of chemical radical-scavenging assays. In vitro assays such as ABTS, DPPH, and FRAP are fundamentally based on artificial, nonphysiologically relevant radicals, and no single method is capable of entirely depicting the oxidative reactions that occur in vivo. Within complex dairy matrices, oxidative reactions are influenced by multiple physicochemical factors. For example, metal ion–catalyzed oxidation can occur when transition metals such as Fe or Cu accelerate the decomposition of lipid hydroperoxides, generating alkoxyl and peroxyl radicals that contribute to the initiation and propagation of lipid oxidation [58]. In addition, oxidative damage in dairy products has been reported to be closely associated with the structural instability or disruption of the milk fat globule membrane (MFGM). When the interfacial membrane acts less effectively as a physical barrier, pro-oxidation substances in the aqueous phase can contact the lipid core more readily, thereby promoting lipid oxidation and subsequent protein oxidation events [59]. Accordingly, the antioxidant capacity of fermented dairy products can be evaluated more reliably when interpreted through an integrated framework that considers (i) chemical-level electron and hydrogen donation properties (ABTS, DPPH, and FRAP), (ii) cell-level changes in ROS and NO production and redox homeostasis, and (iii) organism-level indices of lipid peroxidation damage (TBARS/MDA) alongside modulation of endogenous antioxidant defense systems. Within this context, fermentation-induced peptide release and remodeling of pH and organic acid composition may act as factors that concurrently modulate the initiation and propagation phases of lipid oxidation, representing mechanistic dimensions that cannot be captured by in vitro indices alone. Accordingly, the antioxidant functionality of fermented milk should not be inferred from a single assay outcome but rather described on the basis of concordant evidence across multiple complementary indices that reflect distinct mechanistic dime

## 6. Health Beneficial Effects of Fermented Dairy Products

Table 3 summarizes the microorganisms involved, the observed bioactivities, and the analytical methods used for fermented dairy products. The bioactivities presented in the table can be categorized into (1) metabolic disease–related functionality, (2) inflammation modulation, (3) anticancer activity, and (4) anti-hyperpigmentation effects.

### 6.1. Metabolic Disease-Related Functionality

Fermented milk products have been reported to provide benefits beyond simple antioxidant effects, and are also linked to systemic regulation such as control of chronic inflammation and improvement of the gut microbial environment. A common finding across studies is that intake of fermented milk can reduce pro-inflammatory cytokines and reshape gut microbiota composition, thereby alleviating the inflammation–oxidative stress axis. In a rat model with induced colorectal tumor formation, kefir intake significantly reduced the number of tumors, and this effect was accompanied by lower levels of key inflammatory mediators, including IL-1β, IL-6, TNF-α, and NO [63]. These results suggest that fermented milk may help suppress chronic inflammatory responses that are associated with tumor development.

A similar regulatory pattern was also observed in a functional yogurt produced using the EPS-producing strain *Lactiplantibacillus plantarum* DPA1C. The strain exhibited tolerance to acidic and bile salt conditions, and the resulting yogurt showed α-glucosidase inhibitory activity together with modulation of gut microbiota composition [12]. In particular, a decrease in *Escherichia–Shigella* and an increase in *Bifidobacterium* may be linked to restoration of gut microbial balance, which could contribute to improved inflammatory control and metabolic function.

### 6.2. Inflammation Modulation

Bioactive peptides generated in fermented milk are reported to provide benefits beyond simple antioxidant activity and to exert functionality through specific physiological mechanisms such as blood pressure regulation and control of inflammatory signaling. A common finding across studies is that low-molecular-weight peptides produced during fermentation can act on specific target enzymes involved in blood pressure regulation. ACE-inhibitory peptides produced during milk fermentation by strains such as *L. helveticus* and *L. delbrueckii* subsp. *bulgaricus* were reported to vary in yield and activity depending on the starter strains and milk protein composition, and some of these peptides were shown to remain stable under gastrointestinal conditions [60]. Notably, their antihypertensive effects were supported not only by cell-based ACE inhibition assays but also by evidence from hypertensive animal models and human intervention studies, confirming their physiological relevance in vivo.

Similarly, fatty acid fractions derived from *Lactobacillus*-fermented milk reduced TNF-α and IL-6 levels while increasing IL-10 secretion in LPS-stimulated RAW264.7 cells, which was associated with inhibition of the MAPK and NF-κB signaling pathways [15]. In addition, fermentation of donkey milk increased anti-inflammatory peptides, with peptides below 3 kDa being predominant, suggesting improved bioavailability. These peptides reduced nitric oxide (NO) production in LPS-stimulated RAW264.7 cells. Among them, the peptide HN-17 showed strong binding to inducible nitric oxide synthase (iNOS), and molecular docking suggested that hydrogen bonding and hydrophobic interactions may contribute to enzyme inhibition [17]. Taken together, low-molecular-weight bioactive peptides generated across different fermented milk products may contribute to systemic benefits, including blood pressure control and reduced inflammatory responses, by targeting key regulators such as ACE, NF-κB, and iNOS. This supports the view that fermentation is not simply a general breakdown of proteins, but a process that can selectively generate functional peptide sequences linked to specific signaling pathways.

### 6.3. Anticancer Activity

Reports have also shown that cheese-derived *Lactobacillus* strains can exert selective anticancer effects through protein-based secreted factors. A common finding across studies is that cell-free secretions or protein-based metabolites show low toxicity toward normal cells while selectively inhibiting the growth of cancer cells. *Limosilactobacillus fermentum* C9 and *Lactiplantibacillus plantarum* C47 isolated from traditional cheese inhibited the proliferation of oral cancer cells in a dose- and time-dependent manner, and apoptosis was identified as the main mode of cell death [61]. In particular, increased SMAC expression and decreased SURVIVIN expression were observed, suggesting that the anticancer effect is linked to regulation of apoptosis-related signaling. The authors proposed that these effects may be mediated by protein-based secreted factors.

Similarly, protein-based metabolites from the cheese-derived *Lactiplantibacillus plantarum* Y33 strain were reported to significantly inhibit the survival and proliferation of oral cancer cells [62]. This strain also showed high cell adhesion and strong antimicrobial activity, suggesting a possible link between probiotic traits and anticancer activity. Taken together, the selective inhibition of cancer cells observed for cheese-derived *Lactobacillus* strains may be mediated by protein-based secreted factors and may act through molecular mechanisms that include regulation of apoptosis pathways. These results suggest that protein-based components produced or secreted during fermentation can act as functional bioactive factors rather than being only microbial metabolic products.

### 6.4. Anti-Hyperpigmentation Effects

Whey has been reported to show biological activities related to skin physiology beyond its nutritional components. Whey produced using *Lactobacillus helveticus* (LH-whey) reduced melanin accumulation in B16 melanoma cells, and this effect was associated with decreased expression of key melanin-producing enzymes, including tyrosinase, tyrosinase-related protein-1, and dopachrome tautomerase [16]. These results suggest that components generated during fermentation may regulate the melanogenesis pathway. Interestingly, extracellular vesicles (EVs) isolated from the same whey also showed inhibitory effects on melanin production, whereas no such effect was observed for EVs derived from the corresponding bacterial culture or from milk EVs. This indicates that the melanogenesis-inhibitory activity was specific to the whey-derived EVs in this study.

## 7. Conclusions

In summary, the functional properties of fermented dairy products should be understood as the result of the molecular self-organization of peptides, metabolites, organic acids, and physiologically active factors derived by fermentation within the dairy matrix, rather than being interpreted as a single antioxidant component or a product of individual analysis results. The evidence in this review shows that we can understand how antioxidants really work more accurately if we combine chemical assays (like how well they neutralize free radicals) with other types of evaluations, such as cell-based tests, animal studies, and biomarkers that show how the body defends itself against oxidative stress. Furthermore, this fermentation-induced biochemical reorganization is not limited to antioxidant activity but is also linked to various physiological benefits such as inflammation control, metabolic disease-related functionality, anticancer activity, and skin-related efficacy. Therefore, in the future, fermented dairy products should be more precisely identified through a multi-omics-based approach that considers the specificity of microbial strains, fermentation conditions, and matrix degradation interactions beyond simple antioxidant capacity.

## Figures and Tables

**Table 1:** Representative microorganisms used in fermented milk products and their strain-specific functional effects and proposed mechanisms.

Microorganism	Functional Effect	Strain-Specific Effect	Proposed Mechanism	References
*Lactiplantibacillus plantarum* KU985438, *Lactobacillus rhamnosus* KU985439	Increased antioxidant activity	-KU985438 showed stronger α-amylase inhibition -KU985439 showed stronger hypoglycemic, lipid-modulating, and antioxidant effects.	Suppression of oxidative stress and NF-κB signaling, with modulation of apoptosis-related pathways	[10]
*Lactobacillus helveticus* and *Streptococcus thermophilus*	Enhanced antioxidant peptide activity	-*L. helveticus* showed stronger proteolysis and higher antioxidant and cytoprotective activity. -*S. thermophilus* exhibited better texture and taste but lower antioxidant activity.	Enhanced proteolysis leading to increased low-molecular-weight peptide (<3 kDa) generation	[11]
*Lactiplantibacillus plantarum* DPA1C	Anti-hyperglycemic activity	A high-EPS-producing probiotic strain showing glycemic enzyme inhibitory potential and improved EPS content, viscosity, microbiota profile, and yogurt quality during co-fermentation.	EPS biosynthesis capacity and EPS-mediated modulation of gut microbiota and yogurt quality	[12]
Lactic acid bacteria and acetic acid bacteria	Antioxidant peptide formation	The abundance of *Lactobacillus* was strongly associated with differences in the proteolytic ability of goat milk kefir.	*Lactobacillus*-associated enhancement of casein hydrolysis and proteolysis	[13]
*Limosilactobacillus fermentum* KGL4 and *Saccharomyces cerevisiae* WBS2A	Enhanced radical-scavenging activity	KGL4 is a proteolytically active LAB frequently reported to produce bioactive peptides, while WBS2A is a yeast used in co-culture systems that enhances functional properties.	Proteolysis-driven low-molecular-weight peptide generation, supported by yeast–LAB metabolic interactions	[14]
*Limosilactobacillus reuteri* DSMZ 8533, *Lactiplantibacillus plantarum* A3, and *Lactobacillus acidophilus* CICC 6074	Anti-inflammatory activity	*L. plantarum* A3 showed the strongest anti-inflammatory effect, producing more PUFAs and significantly reducing TNF-α and IL-6 while increasing IL-10. *L. acidophilus* CICC 6074 also increased PUFAs and IL-10, but to a lesser extent. In contrast, *L. reuteri* DSMZ 8533 showed lower fatty acid levels and PUFA enrichment, indicating weaker functional potential under the tested conditions.	Modulation of lipase activity, PUFA biosynthesis, and inflammation-related signaling pathways	[15]
*Lactobacillus helveticus* JCM1120	Antihyperpigmentation effect	Showed the strongest anti-melanogenic activity among the tested strains and reduced melanin production and TYR expression.	Suppression of melanogenesis enzymes and autophagy-associated melanosome degradation mediated by whey-derived EVs	[16]
*Lactiplantibacillus plantarum* 350-2 and *Lactobacillus bulgaricus* SH34-3	Anti-inflammatory activity	*L. plantarum* 350-2 showed the strongest proteolytic activity among the tested strains and was selected as a starter strain, whereas *L. bulgaricus* SH34-3 showed the second-highest proteolytic activity.	Proteolysis-driven peptide remodeling and anti-inflammatory peptide generation	[17]

**Table 2:** Antioxidant capacities and bioactivities of fermented milk products.

Dairy Product	Microorganism	Antioxidant Capacity and Bioactivity	Evidence Level	References
Yogurt	*Lactiplantibacillus plantarum* KU985438, *Lactobacillus rhamnosus* KU985439	-After 15 days of feeding the fermented yogurt to STZ-induced diabetic rats, fasting blood glucose decreased from 309.3 ± 11.0 to 164.0 ± 7.0 and 155.1 ± 9.6 mg/dL, hepatic MDA decreased from 42.1 to 38.92/32.6 nmol/g, and GSH increased from 9.3 to 10.0/10.1 mg/g, indicating enhanced antioxidant capacity.	Cell models	[10]
Yogurt	*Streptococcus thermophilus*, *Lactobacillus delbrueckii* subsp. *bulgaricus*	-Reduced LDL-C was observed in the high-fat-diet-fed rats supplemented with yogurt. -A decrease in the inflammatory marker C-reactive protein was reported. -SCFAs (butyrate) may have anti-inflammatory and antioxidant potential.	Animal models	[31]
Cheese	*Lactobacillus helveticus*, *Streptococcus thermophilus*	-In a cell injury model, ROS and MDA levels were reduced while CAT (catalase activity) and SOD (superoxide dismutase activity) activities were increased. -Four peptides (AMKP-WIQPK, AVPYPQR, EMPFPK, and VLPVPQK) were identified in the highest antioxidant activity fraction	Cell models	[11]
Kefir	Lactic acid bacteria (LAB), acetic acid bacteria (AAB)	-Kefir-derived phosphopeptides were mainly generated from β-casein (79% of identified phosphopeptides), with minor contributions from αs1-casein (8%) and αs2-casein (9%), and 71 of 73 peptides (97%) contained the SerP-SerP-SerP-Glu-Glu calcium-binding motif.	In vitro assays	[13]
Kefir	*Lactobacillus kefiranofaciens*, *Lactobacillus kefiri*, *Saccharomyces*, *Kazachstania*, *Kluyveromyces*	-Peptides were predominantly derived from β-casein. -β-Lactoglobulin was relatively resistant to microbial hydrolysis. -β-Casein–derived peptides with ACE-inhibitory and antioxidant activities were detected.	In vitro assays	[32]
Kefir	*Lactobacillus* spp., *Acetobacter* spp.	-Administration of kefir peptide fractions (WSF, ≥10 kDa, and <10 kDa) at 0.25 or 0.5 mg/mL for 10 days in AD-like Drosophila resulted in a significant reduction in brain β-amyloid levels, as assessed by Thioflavin T fluorescence, and in the neurodegeneration index compared to the control group, alongside an improvement in oxidative stress parameters measured by the FRAP assay; notably, treatment with the <10 kDa fraction at 0.25 mg/mL also led to a significant enhancement of negative geotaxis performance (climbing ability) and a significant decrease in acetylcholinesterase activity.	Animal model	[33]
Koumiss	*Lactobacillus delbrueckii* subsp. *bulgaricus*, *Streptococcus thermophilus*, *Lacticaseibacillus casei*, *Bifidobacterium lactis*	-Changes in functional compounds such as p-pyruvate, 20-HETE, 4-aminobutanoate, uracil, and acetoacetate were identified.	In vitro assays	[34]
Sheep milk	*Limosilactobacillus fermentum* KGL4 (MTCC 25515), *Saccharomyces cerevisiae* WBS2A (MG101828)	-ABTS radical-scavenging activity reached up to 40.08 ± 0.53%. -In RAW 264.7 cells, treatment with fermented sheep milk reduced intracellular ROS from 44% to 11.7%.	In vitro assays + Cell models	[14]
Probiotic fermented milk	*Lacticaseibacillus paracasei* PC-01, *Bifidobacterium adolescentis* B8589	-Increased GABA -Production of succinic acid	In vitro assays	[35]
Fermented cow’s milk	*Lactiplantibacillus plantarum* (*ropy phenotype* T1, *non-ropy phenotype* CSK)	-Fermented milk produced with *L. plantarum* T1 showed scavenging activities of 77.49% for ABTS, 42.11% for DPPH, and 49.44% for hydroxyl radicals, whereas CSK-fermented milk showed 71.52%, 38.12%, and 41.46%, respectively. -Overall increases in amino acids, peptides, vitamins, cofactors, and organic acids were observed.	In vitro assays	[36]
Binglangjiang buffalo fermented milk (BBFM)	*Lacticaseibacillus casei Zhang*, *Lactobacillus delbrueckii* subsp. *bulgaricus*, *Streptococcus thermophilus*	-Anti-inflammatory peptide activity derived from BBFM was investigated. -A total of 769 peptides were identified. -GPGAPADPGRPTG (GG13) was selected. -GG13 was confirmed to significantly inhibit NO and TNF-α secretion in cells.	Cell models	[37]

**Table 3:** Health benefits of fermented milk products.

Dairy Product	Microorganism	Bioactivity	Analytical Method	Evidence Level	References
LAB fermented milk	*Lactobacillus helveticus*, *Lactobacillus delbrueckii* subsp. *bulgaricus*	-ACE-inhibitory peptides reduce serum ACE activity and angiotensin II production. -The ACE inhibitory IC_50_ of peptides was reported to be as low as approximately 5 μM.	-ACE inhibition assay -ELISA	In vitro assays	[60]
Yogurt	*Lactiplantibacillus plantarum* DPA1C	-Anti-hyperglycemic functionality via reduced α-glucosidase activity -Gut microbiota modulation -It showed an EPS content of 45.13 mg/kg and an in vitro α-glucosidase inhibition rate of 54.01%.	-16s rRNA amplicon sequencing -Whole genome sequencing -UV–Vis spectrophotometry–based assays -HS-SPME/GC–MS	In vitro assays	[12]
LAB fermented milk	*Limosilactobacillus reuteri* DMSZ 8533 *Lactiplantibacillus plantarum* A3, *Lactobacillus acidophilus* CICC 6074	-Hydrolyzed milk fat lipids attenuate inflammation by suppressing TNF-α and IL-6 and enhancing IL-10 via the MAPK–NF-κB pathway. -The anti-inflammatory activity of fermented milk fatty acid profiles was confirmed in LPS-stimulated RAW264.7 cells. -Treatment with 100 μmol of fermented milk–derived fatty acids (including ALA as a positive control) reduced TNF-α levels in LPS-stimulated RAW264.7 cells to approximately 558 pg/mL, accompanied by a significant decrease in IL-6 and a significant increase in IL-10.	-Fatty acid profiling (GC-based analysis) -Cell culture model: LPS-induced RAW264.7 macrophages -qPCR/ELISA for inflammatory cytokines -Pathway analysis (MAPK/NF-κB signaling)	Cell models	[15]
Cheese	*Lactobacillus* spp., *Lactococcus* spp., *Streptococcus* spp.	-Using the well-diffusion method, the *Lactobacillus* strains C9 and C47 showed significant inhibitory effects against human pathogenic microorganisms. -An MTT assay demonstrated that compounds extracted from strains C9 and C47 suppressed the survival and growth of KB and OSCC oral cancer cells. -At 25 μg/mL (72 h), C9 and C47 reduced KB and OSCC cell viability to ~8–18% (≈82–93% growth inhibition).	-MTT assay -qRT-PCR analysis	Cell models	[61]
*Lactobacillus helveticus* (LH-whey)	*Lactobacillus helveticus* JCM1120	-Inhibition of α-MSH–induced melanogenesis (anti-hyperpigmentation effect) -In B16 cells, α-MSH increased melanin content to approximately 175–180% of control, whereas LH-whey reduced this to about 115–120%, corresponding to an inhibition of roughly 32–35% versus the α-MSH-treated group.	-B16 melanoma cell assay -RT–PCR-Western blot	Cell models	[16]
Milk, Cheese, Yogurt	*Lactobacillus* spp.	-Y33 isolated from milk, yogurt, and cheese exhibited strong probiotic potential -Inhibition of oral cancer cell proliferation -Treatment with 20 µg/mL of secreted metabolites from *Lactiplantibacillus plantarum* Y33 reduced the viability of KB and OSCC oral cancer cells to below 26% after 72 h, with little to no significant cytotoxicity observed in normal fibroblast and HUVEC cells up to 48 h under the same conditions.	-Agar well diffusion assay -MTT cell viability assay -AO/EB fluorescent staining + fluorescence microscopy -qRT PCR -Simulated GI tolerance test	Cell models	[62]
Donkey milk	*Lactiplantibacillus plantarum* 350–2, *Lactobacillus bulgaricus* SH34–3	-A higher proportion of anti-inflammatory peptides was observed in fermented donkey milk than in raw donkey milk. -Peptides derived from fermented donkey milk effectively reduced nitric oxide (NO) production in RAW264.7 cells. -In RAW264.7 macrophages, treatment with FDM-derived <3 kDa peptides at 25, 50, and 100 µg/mL resulted in cell viabilities of 89.62 ± 1.44%, 100.33 ± 2.46%, and 96.61 ± 2.83%, indicating minimal cytotoxicity. In contrast, 2 µg/mL LPS increased NO levels from 4.36 ± 0.06 to 28.96 ± 0.36 µmol/L, but subsequent treatment with the same peptide concentrations significantly reduced NO levels to 23.80 ± 0.12, 23.07 ± 0.15, and 22.30 ± 0.11 µmol/L, respectively, suggesting that these peptides effectively suppressed inflammation-related NO production while preserving cell viability.	-NanoLC-DIA-HRMS/MS	Cell models	[17]
Kefir	*Lactococcus* spp., *Lactobacillus* spp.	-Reduced incidence and number of colorectal tumors -Decreased IL-1β, IL-6, TNF-α, IFN-γ, and NO -Gut microbiota modulation: increased protective taxa and decreased pro-inflammatory taxa -Compared with the normal diet control group (NL), the overfed obese group (SL) showed a 21.78% increase in final body weight, a 53.83% increase in visceral adiposity, and 100% tumor incidence with 2.0 ± 0.57 tumors per animal. Kefir supplementation suppressed tumor formation, with no tumors detected in KNL and a 71.43% reduction in tumor number in KSL. It also reduced colonic inflammatory markers, including IL-1β, TNF-α, NO, and IL-6.	-16S rRNA gene sequencing -ELISA (IL-1β, IL-6, TNF-α, IFN-γ) -Griess assay (Nitric oxide) -Histopathology (colon tissue) -Tumor incidence/count	Animal models	[63]

## Data Availability

No new data were created or analyzed in this study.
